# Loss and reduced expression of PTEN correlate with advanced-stage gastric carcinoma

**DOI:** 10.3892/etm.2012.749

**Published:** 2012-10-15

**Authors:** XUEHUA ZHU, XIA QIN, MAOGUI FEI, WENMIN HOU, JOEL GRESHOCK, KURTIS E. BACHMAN, JIUHONG KANG, CRYSTAL YING QIN

**Affiliations:** 1 Shanghai Key Laboratory of Signaling and Disease Research, School of Life Science and Technology, Tongji University;; 2 Department of Oncology, GlaxoSmithKline Research and Development Center;; 3 Institute for Nutritional Sciences, Shanghai Institutes for Biological Sciences, Graduate School of the Chinese Academy of Sciences (CAS), Shanghai, P.R. China;; 4 Cancer Research, GlaxoSmithKline, Collegeville, PA, USA

**Keywords:** phosphatase and tensin homolog, advanced gastric carcinoma, tissue microarray, immunohistochemistry, receiver operator characteristic curve

## Abstract

Phosphatase and tensin homolog (PTEN) is a tumor suppressor involved in multiple cell processes. To investigate the role of PTEN in the development of gastric carcinoma, we determined the expression pattern of PTEN in primary gastric carcinoma and in paired adjacent non-neoplastic tissue. We also determined the correlation of PTEN expression with clinicopathological characteristics and patient survival. Overall, 159 gastric carcinomas and 151 paired adjacent non-neoplastic tissues were used in the present study. PTEN expression was determined using tissue microarrays and immunohistochemistry. The clinical sensitivity and specificity of PTEN expression were calculated using receiver operator characteristic curves. Results showed that the loss of cytoplasmic PTEN was significantly more frequent in carcinoma tissue compared with adjacent non-neoplastic tissue (62 vs. 5%, respectively; P<0.0001). PTEN expression was markedly downregulated in carcinoma tissues compared with adjacent non-neoplastic tissues. The loss of cytoplasmic PTEN expression was positively correlated with histological stage (P=0.016). The loss of nuclear or total PTEN, and downregulation of total PTEN expression, was significantly different between American Joint Committee on Cancer tumors of stage I and stages II–IV. A low cytoplasmic or total PTEN expression showed high clinical sensitivity and specificity for gastric carcinoma. However, PTEN expression was not significantly associated with overall or 3-year survival rates. The findings of the present study indicated that PTEN expression may be a molecular diagnostic marker for gastric cancer. Thus, the loss or reduced expression of PTEN potentially correlate with advanced stages of gastric carcinoma.

## Introduction

Gastric carcinoma is one of the most common types of cancer affecting the digestive system. Over one-third of all gastric carcinoma cases worldwide occur in China (1,2), where the incidence and mortality rates of gastric carcinoma are on the increase. The 5-year survival rate of patients with gastric cancer is currently less than 20%, since most patients are diagnosed at a later stage with advanced disease, when metastasis has occurred, and are therefore often unsuitable for curative surgery. Metastasis is the main cause of treatment failure in patients with gastric carcinoma. Considering the difficulty of treating disseminated disease, which is often apparent at diagnosis, as well as the poor prognosis of patients with gastric carcinoma, molecular diagnostic and prognostic markers for this aggressive form of cancer should be established.

Phosphatase and tensin homolog (PTEN) is a tumor suppressor, also known as mutated in multiple advanced cancer 1 or tumor growth factor-β-regulated and epithelial cell-enriched phosphatase 1. Its gene is located on chromosome 10q 23.3 (3–5) and encodes a 403-residue protein with lipid and protein phosphatase activity (6). PTEN, functioning as a lipid phosphatase, dephosphorylates phosphatidylinositol-(3,4,5)-triphosphate (PIP3) (6), a lipid product of class I phosphoinositide 3-kinases. In turn, PIP3 activates important kinases such as phosphoinositide-dependent kinase 1 and the serine-threonine protein kinase AKT (7). Therefore, PTEN affects processes such as cell cycle progression, apoptosis, migration, metabolism, transcription and translation by negatively regulating the AKT pathway and decreasing phosphorylation of AKT substrates (8). PTEN, functioning as a protein phosphatase, is able to dephosphorylate focal adhesion kinase, which inhibits cell invasion and metastasis. PTEN also inhibits the mitogen-activated protein kinase signaling pathway, thus restricting cell differentiation (9,10).

The loss or downregulation of PTEN appears to be a common event in many types of tumors. The PTEN gene was previously reported to be transcriptionally silenced by promoter methylation in a number of gastric cancer cases (11). However, the role of the loss or reduced expression of PTEN in gastric carcinoma progression and prognosis remains unclear, especially when including paired adjacent non-neoplastic tissue as a control. Since changes of gene expression in a tumor may be due to individual variation rather than tumor-specific activity, paired adjacent non-neoplastic tissue samples were obtained to investigate for possible variations in PTEN expression among patients.

The aim of this study was to evaluate the clinical significance of the expression of PTEN protein in patients with gastric carcinoma, and to investigate the correlation of PTEN expression with the clinicopathological parameters and the prognosis of these patients.

## Materials and methods

### Tissue microarray

The gastric cancer (GC) tissue microar-rays (TMAs) used in the present study were purchased from Shanghai Outdo Biotech Co., Ltd. (Shanghai, China). Human research ethics was approved by the Ethics Committee of the Third Xiangya Hospital, Central South University (Hunan, China). All patients provided written informed consent to participate in this study. In total, 159 tumor tissues and 151 paired adjacent non-neoplastic tissues were obtained. None of the 159 patients had received radiotherapy or chemotherapy prior to surgery. Clinicopathological data including gender, age at diagnosis, histological grade, American Joint Committee on Cancer (AJCC) tumor stage, depth of invasion, lymph node metastasis, distant metastasis and clinical follow-up information were obtained from all patients.

Gastric carcinoma samples were histologically reviewed by one pathologist. Tumors were graded according to the Thoenes grading system and were histologically classified according to the World Health Organization Classification System. Depth of invasion and lymph node metastasis were staged according to the Union for International Cancer Control (UICC) criteria. Patients with lymph node metastasis stage >N0 or with distant metastasis (i.e., M1) were considered to have metastatic disease. Overall survival time was estimated as the time from diagnosis to the date of death or last contact.

### Immunohistochemistry

Immunostaining of the TMA slides was performed on a TechMate 500 (Dako A/S, Copenhagen, Denmark) automatic staining instrument according to the manufacturer’s instructions. The TMA slides were incubated with PTEN monoclonal antibody (dilution, 1:50; #9559, clone 138G6, Cell Signaling Technology, Inc., Beverly, MA, USA) overnight at 4°C. The TMA slides were then incubated for 30 min with a labeled polymer horseradish peroxidase detection kit (EnVision+; Dako, Carpinteria, CA, USA). A positive control, derived from tissue with previously confirmed PTEN expression, was used in all TMAs. Slides in which the PTEN antibody replaced by control IgG (#3900, Cell Signaling Technology) served as negative controls. Signal detection was performed using a Dako signaling amplification system.

### Evaluation of tissue staining

PTEN expression was evaluated according to the staining intensity and the percentage of cells expressing PTEN. PTEN staining was evaluated by one pathologist and two observers simultaneously, and a consensus was reached for each score. Tissues with <5% of PTEN-positive cells were labeled 0. Staining intensity was scored from 0 to 3 (0, negative; 1, weak; 2, moderate and 3, strong). The level of PTEN staining was evaluated by calculating the immunoreactive score (IRS) (12) from the staining intensity (I) and the percentage (P) of PTEN-positive cells: IRS=IxP. IRS=0 was considered as negative and IRS>0 as positive expression.

Total PTEN expression was calculated as cytoplasmic PTEN expression + nuclear PTEN expression. Cases with total IRS of gastric carcinoma tissues/total IRS of paired adjacent non-neoplastic tissues ≤0.5 were considered to have a down-regulated PTEN expression.

### Statistical analysis

Analyses were performed using GraphPad Prism software version 5 (GraphPad Software, Inc., San Diego, CA, USA) for Windows and MedCalc software (MedCalc, Mariakerke, Belgium). The Student’s t-test was used to compare PTEN expression between tumor and adjacent non-neoplastic tissue. The correlations between PTEN expression and clinicopathological characteristics were analyzed using contingency tables and Pearson’s χ^2^ test, except for parameters with small sample sizes, for which Fisher’s exact test was used. Survival time was analyzed according to the PTEN expression level by the Kaplan-Meier method and compared using the log-rank test. Cox regression model was used for multivariate analyses. The clinical sensitivity and specificity of PTEN expression were determined using receiver operator characteristic (ROC) curves, and area under the curve (AUC) was calculated. In all analyses, P<0.05 was considered to indicate a statistically significant difference.

## Results

### Patient clinicopathological characteristics

The clinicopathological characteristics of the 159 *de novo* gastric carcinoma patients are recorded in Table I. There were 112 males with a median age of 63 years (range, 45–84), and 47 females with a median age of 67 years (range, 34–83). Histological grade was used to assess differentiation stage; 2, 40 and 117 patients had grade I, II and III tumors, respectively. AJCC tumor stage was used to stage the primary gastric carcinomas; 13, 49, 86 and 11 patients were classified as having stage I, II, III and IV tumors, respectively. Depth of invasion was assessed using UICC criteria, and 11, 14, 104 and 30 patients were classified as stage T1, T2, T3 and T4, respectively. Lymph node metastasis was assessed using UICC criteria and 36, 26, 49 and 48 patients were classified as N0, N1, N2 and N3, respectively. Regarding distant metastasis, 148 patients were classified as M0, and 11 as M1. The mean duration of follow-up was 33.7 months (median, 38 months; range, 0–61 months).

### Gastric carcinomas showed significant loss of cytoplasmic but not nuclear PTEN expression relative to non-neoplastic tissues

**Figure 1:**
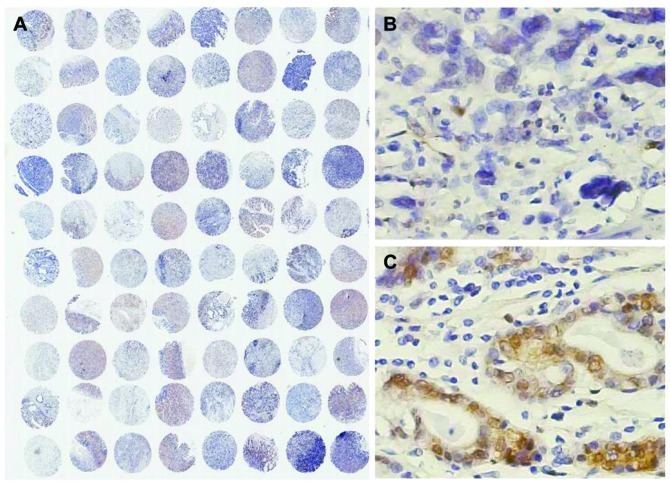
Immunohistochemical staining of PTEN protein is shown. (A) A typical tissue microarray. Each cylindrical core represents a tumor tissue specimen or an adjacent non-neoplastic tissue specimen. (B and C) Representative images of gastric carcinoma tissues showing (B) negative or (C) positive PTEN expression.

**Figure 2:**
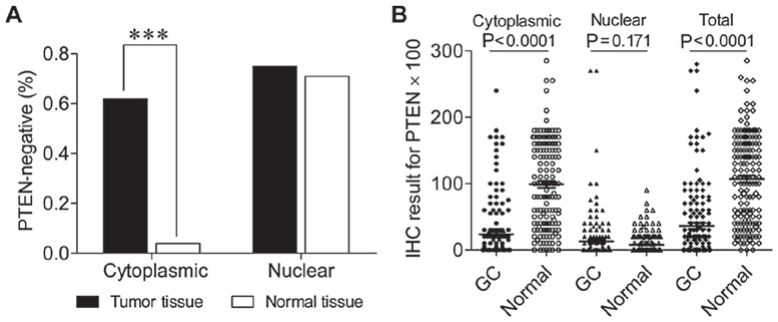
PTEN expression is shown. (A) Proportion of gastric carcinoma and adjacent non-neoplastic tissues negatively stained for PTEN. ^***^P<0.0001. (B) Immunohistochemical (IHC) staining scores for cytoplasmic, nuclear and total PTEN expression in gastric carcinoma (GC, closed symbols) and adjacent non-neoplastic tissues (normal, open symbols). Circles, cytoplasmic expression; triangles, nuclear expression; diamonds, total expression.

The expression levels of PTEN in gastric carcinoma and adjacent non-neoplastic tissue were determined by immuno histochemistry using a PTEN-specific antibody (Fig. 1A). The immunostaining pattern of PTEN was characterized by cytoplasmic and nuclear staining of the carcinoma and adjacent non-neoplastic tissues. Representative images of PTEN-negative and -positive expression are shown in Fig. 1B and C, respectively. The percentage of gastric carcinoma tissue samples lacking cytoplasmic PTEN expression (62%, 98/159) was significantly higher compared with that of adjacent non-neoplastic tissue (5%, 7/151) (Fig. 2). By contrast, the percentages of gastric carcinoma tissue and adjacent non-neoplastic tissue samples lacking nuclear PTEN expression were high, but similar (75%, 119/159 vs. 70%, 105/151, respectively) (Fig. 2A). Quantification of immunohistochemistry, confirmed that cytoplasmic and total PTEN expression levels were significantly lower in gastric carcinoma tissues compared to adjacent non-neoplastic tissues (both, P<0.0001) (Fig. 2B), no difference was detected in nuclear expression (P=0.171). These data indicate that, relative to adjacent non-neoplastic tissue, the loss of PTEN expression in gastric carcinoma is mainly due to the downregulation or loss of cytoplasmic rather than nuclear expression.

### Correlations between loss of cytoplasmic/nuclear PTEN expression and clinicopathological characteristics

**Figure 3:**
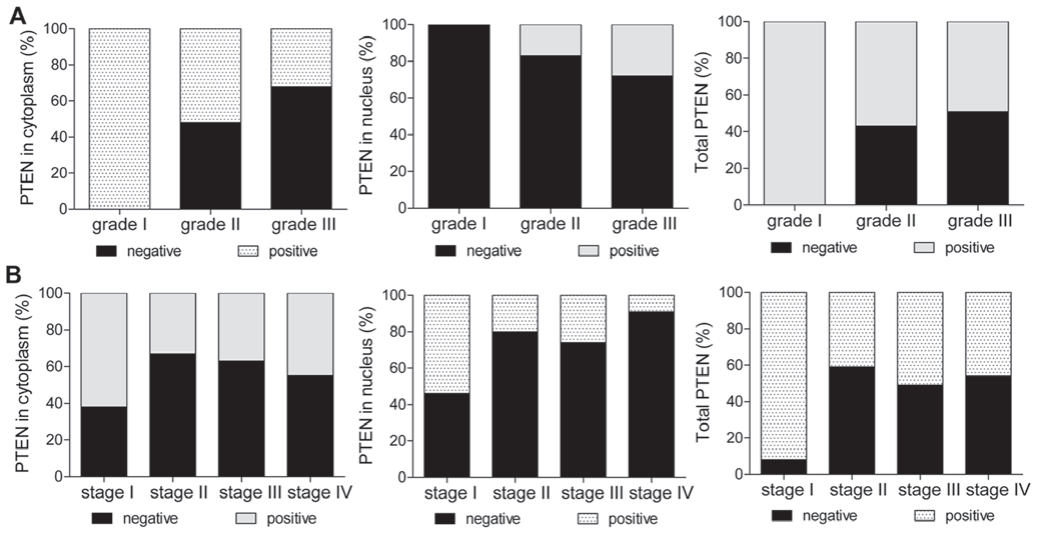
The correlation between the loss of PTEN expression and (A) histological grade or (B) AJCC stage is shown. (A) The loss of cytoplasmic PTEN expression (P= 0.016), but not nuclear or total expression, was significantly correlated with histological grade. (B) The loss of total PTEN expression was significantly correlated with AJCC stage (P= 0.012). The loss of nuclear (P= 0.013) and total (P= 0.002) PTEN expression was also significantly different between AJCC tumor stage I and stages II–IV. AJCC, American Joint Committee on Cancer.

Among 159 patients with gastric carcinoma, the histological grade was significantly correlated with the loss of cytoplasmic PTEN expression (P=0.016) (Fig. 3A and Table I), but not with the loss of nuclear or total PTEN expression (Fig. 3A). AJCC tumor stage was significantly correlated with the loss of nuclear PTEN expression (P=0.013, AJCC tumor stage I vs. stages II–IV) and with the loss of total PTEN expression (P=0.012) (Fig. 3B and Table I). When comparing AJCC tumor stage, the percentage of stage II–IV carcinomas with loss of total PTEN expression was significantly greater compared with that of stage I carcinomas (P=0.002) (Fig. 3B and Table I). These results suggest that the loss of total PTEN expression is an effective marker which may be used in the differentiation of AJCC stage I from stage II–IV tumors. No statistically significant correlations between the loss of cytoplasmic, nuclear or total PTEN expression with other clinicopathological characteristics, including gender, age, depth of invasion, lymph node metastasis and distant metastasis were observed (Table I).

### Correlation between the downregulation of PTEN expression and clinicopathological characteristics

**Figure 4:**
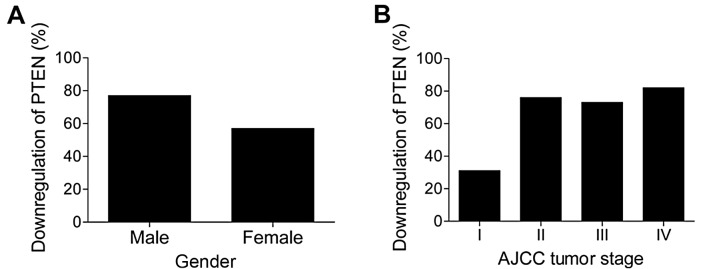
The correlation between downregulated total PTEN expression and clinicopathological characteristics is shown. Downregulated total PTEN expression in gastric carcinoma tissues was significantly associated with (A) gender (P=0.025) and (B) AJCC tumor stage (P=0.018). The correlation between PTEN expression and AJCC stage was more significant when comparing AJCC tumor stage I with stages II–IV (P=0.004). AJCC, American Joint Committee on Cancer.

Possible correlations were investigated between the downregulation of total PTEN expression (i.e., total IRS of gastric carcinoma tissues/total IRS of paired adjacent non-neoplastic tissues ≤0.5) with the clinicopathological characteristics of gastric carcinoma patients. Downregulation of PTEN expression was significantly correlated with gender (P=0.025) (Fig. 4A and Table I) and AJCC tumor stage (P= 0.018) (Fig. 4B and Table I). When AJCC stage I carcinomas were compared with stage II–IV carcinomas, the association between the downregulation of PTEN expression and AJCC tumor stage became more significant (P=0.004) (Fig. 4B and Table I), suggesting that the downregulation of PTEN has the potential to be used in the differentiation of AJCC stage I from stage II–IV tumors. By contrast, no correlation between the downregulation of total PTEN expression with other clinicopathological characteristics was observed (Table I).

### Clinical sensitivity and specificity of PTEN expression

**Figure 5:**
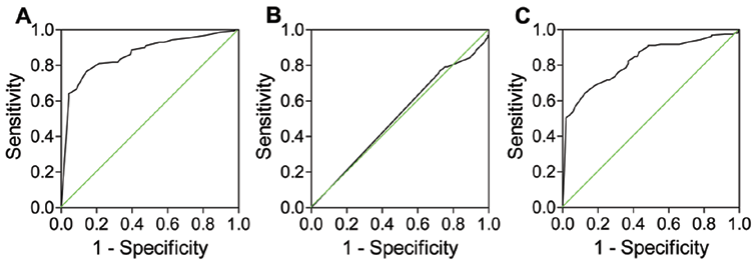
Receiver operator characteristic (ROC) curves for the clinical sensitivity and specificity of (A) cytoplasmic, (B) nuclear and (C) total PTEN expression in gastric carcinoma relative to adjacent non-neoplastic tissue. The area under the curve (AUC) represents the overall accuracy (range, 0–1.0). The green line represents the accuracy achieved by chance alone (AUC=0.5).

The clinical sensitivity and specificity of PTEN expression were examined using ROC curve analysis and the AUC was calculated as an indicator of overall discrimination. The AUCs for cytoplasmic, nuclear and total PTEN expression in gastric carcinoma were 0.865 (P<0.0001), 0.516 (P=0.617) and 0.829 (P<0.0001), respectively (Fig. 5 and Table II). When using the optimal cut-off point determined by MedCalc software, the diagnostic accuracies of cytoplasmic, nuclear and total PTEN expression were 85.9, 53.3 and 85.7%, respectively. Thus, a low PTEN cytoplasmic or total expression showed significant clinical sensitivity and specificity in gastric carcinoma, and was able to differentiate between carcinoma tissue and adjacent non-neoplastic tissue.

### Correlation between loss of PTEN expression and survival

In order to evaluate the prognostic relevance of PTEN expression in patients with gastric cancer after surgery, the Cox regression model was used to investigate the effects of PTEN expression on overall and 3-year survival. As shown in Tables III and IV, cytoplasmic, nuclear and total PTEN expression was not associated with median survival time or overall survival. These data indicate that PTEN expression is not associated with overall or 3-year survival of patients with gastric carcinoma after surgery.

## Discussion

In the present study, we used TMAs of tumor and paired adjacent non-neoplastic tissues to evaluate the clinical significance of PTEN in patients with gastric carcinoma. We showed that PTEN expression was frequently lost in the cytoplasm in gastric carcinoma compared with adjacent non-neoplastic tissue. The loss of cytoplasmic PTEN expression was significantly correlated with histological grade, and the loss of nuclear or total PTEN expression was significantly correlated with AJCC tumor stage. The level of PTEN expression was also downregulated in gastric carcinoma compared with paired adjacent non-neoplastic tissue. Downregulation of total PTEN expression was significantly associated with gender and AJCC tumor stage, and the frequency of PTEN downregulation was positively correlated with AJCC tumor stage. Thus, a low PTEN expression may be a marker for gastric carcinoma. These findings also indicate a novel molecular basis for the critical role of PTEN loss in the development and progression of gastric carcinoma.

The tumor suppressor PTEN is encoded by a gene that shows the greatest selection for loss in the human genome (13). Studies have shown that the PTEN gene is frequently mutated or lost in many types of human primary carcinomas (14). In addition, PTEN expression is often dysregulated in carcinoma, even in the absence of genetic loss or mutation (15). In mice, PTEN deletion or mutation significantly contribute to tumorigenesis, and conditional knockout of PTEN leads to neoplasia in multiple tissues (16,17). Functional PTEN expression has been shown to inhibit the growth and invasive properties of cancer cells, and thus improve survival outcomes in various types of tumors (18–22). These studies have demonstrated the pivotal roles of PTEN in cancer initiation and progression. Similarly, we found that 62% of the gastric carcinomas demonstrated loss of cytoplasmic PTEN compared with 5% of adjacent non-neoplastic tissues, by using TMA and immuno histochemistry. Moreover, 72% of the gastric carcinomas showed downregulation of total PTEN expression relative to the adjacent non-neoplastic tissues. The lower cytoplasmic and total PTEN expression levels in gastric carcinoma compared to adjacent non-neoplastic tissues observed in this study were consistent with the findings reported by Zheng *et al*(23). Furthermore, the reduced cytoplasmic or total PTEN expression levels had significant clinical implications for gastric carcinoma based on ROC curves. These data indicate that downregulation or loss of PTEN may be an etiological factor in the development and progression of gastric carcinoma.

In mice, tumor burden and levels of phosphorylated AKT increase significantly when the expression level of PTEN decreases by 25%, particularly in the mammary gland (24). These findings suggest that even small reductions in PTEN catalytic activity are likely to have a significant clinical impact. In the gastric carcinoma patients included in this study, the rate of loss of cytoplasmic PTEN expression was positively correlated with histological grade. The loss or downregulation of total PTEN expression was also correlated with AJCC tumor stage. Thus, the loss of nuclear or total PTEN expression, and downregulation of total PTEN expression can be used in the differentiation of AJCC stage I from stage II–IV gastric carcinomas. These data also suggest that PTEN expression is significantly associated with the progression of gastric carcinoma.

A number of studies have shown that a decreased PTEN expression is also correlated with the progressive outcome of solid cancers, including ovarian, prostate and cervical cancer (25,26). Regarding its association with survival, Deng *et al*(27) reported that PTEN expression was not correlated with survival time, whereas Bai *et al*(28) showed that patients with nuclear PTEN expression had higher survival rates compared with those without nuclear PTEN expression. In this study, we found no significant correlation of PTEN expression, including cytoplasmic, nuclear and total expression, with overall or 3-year survival, results which are consistent with the findings of Deng *et al*(27). Although the differences in associations observed between our study and the results reported by Bai *et al*(28) may be due to methodological differences or the number of patients examined, it is likely that PTEN expression in gastric carcinoma may not serve as a prognostic marker, but as a marker for differentiating tumor stage and progression. Additional studies are required to further explore the role of PTEN expression (or lack thereof) in the prognosis of gastric cancer, and take into account postsurgical treatments that potentially affect survival independently of PTEN expression, such as adjuvant therapy.

In conclusion, results showed that the cytoplasmic PTEN expression was frequently lost in gastric carcinoma compared with adjacent non-neoplastic tissue. Furthermore, the total PTEN expression was downregulated in gastric carcinoma relative to non-neoplastic tissue in the patients included in this study. Loss and downregulation of PTEN expression were associated with several clinicopathological characteristics, notably AJCC stage. These findings suggest that the loss or downregulation of PTEN expression is involved in tumorigenesis and the progression of primary gastric carcinoma. Our findings also indicate that PTEN is a promising new molecular target for designing novel preventive and therapeutic strategies to control gastric carcinoma.

## Figures and Tables

**Table I:** Clinicopathological characteristics of 159 gastric cancer patients according to PTEN expression profile.

Characteristics	Total no.	Cytoplasmic PTEN negative, n=98 (62%)	P value	Nuclear PTEN negative, n=119 (75%)	P value	Total PTEN negative, n=77 (48%)	P value	Downregulated PTEN expression, n=113 (71%)	P value
Gender									
M	112	70 (63%)	0.725	84 (75%)	1	54 (48%)	1	82 (78%)	0.025
F	47	28 (60%)		35 (74%)		23 (49%)		25 (58%)	
Age (years)									
Median	65	65		67		65		65	
Range	34–84	41–83		41–84		41–83		41–84	
Histological grade									
I	2	0 (0%)	0.016	2 (100%)	0.287	0 (0%)	0.244	1 (50%)	0.717
II	40	19 (48%)		33 (83%)		17 (43%)		28 (70%)	
III	117	79 (68%)		84 (72%)		60 (51%)		80 (73%)	
AJCC stage									
I	13	5 (38%)	0.271	6 (46%)	0.051	1 (8%)	0.012	4 (33%)	0.018
II	49	33 (67%)		39 (80%)	0.013^a^	29 (59%)	0.002^a^	36 (77%)	0.004^a^
III	86	54 (63%)		64 (74%)		42 (49%)		60 (74%)	
IV	11	6 (55%)		10 (91%)		5 (45%)		9 (82%)	
Depth of invasion									
T1	11	7 (64%)	0.897	7 (64%)	0.828	4 (36%)	0.851	6 (60%)	0.578
T2	14	8 (57%)		11 (79%)		7 (50%)		10 (71%)	
T3	104	66 (63%)		78 (75%)		52 (50%)		71 (71%)	
T4	30	17 (57%)		23 (77%)		14 (47%)		22 (81%)	
Lymph node metastasis									
N0	36	20 (56%)	0.836	27 (75%)	0.632	17 (47%)	0.841	22 (65%)	0.213
N1	26	16 (62%)		17 (65%)		11 (42%)		17 (68%)	
N2	49	32 (65%)		37 (76%)		26 (53%)		40 (83%)	
N3	48	30 (63%)		38 (79%)		23 (48%)		30 (68%)	
Distant metastasis									
M0	148	92 (62%)	0.75	109 (74%)	0.29	72 (49%)	1	100 (71%)	0.728
M1	11	6 (55%)		10 (91%)		5 (45%)		9 (82%)	

a AJCC stage I vs. stages II–IV. M, male; F, female; AJCC, American Joint Committee on Cancer.

**Table II:** Receiver operating characteristic curve analysis of PTEN expression.

			Optimal cut-off point						
PTEN expression pattern	AUC (95% CI)	P-value	Sensitivity (%)	Specificity (%)	+PV (%)	−PV (%)	+LR	+LR	Diagnostic accuracy (%)
Cytoplasmic	0.865 (0.822–0.901)	<0.0001	76.1	87.4	85.9	77.4	6.05	0.27	85.9
Nuclear	0.516 (0.459–0.573)	0.617	76.7	29.1	53.3	54.3	1.08	0.80	53.3
Total	0.829 (0.783–0.870)	<0.0001	64.2	88.7	85.7	70.2	5.7	0.40	85.7

The optimal cut-off point was defined using MedCalc software. AUC, area under the curve; CI, confidence interval; PV, predictive value; LR, likelihood ratio.

**Table III:** Correlation of PTEN expression with overall survival.

PTEN expression profile	HR (95% CI)	P-value	Median survival time (months)	Survival rate (%)
Cytoplasmic				
Negative	0.856 (0.556–1.319)	0.481	43	43
Positive			28	41
Nuclear				
Negative	1.126 (0.704–1.804)	0.62	37	42
Positive			43	43
Total				
Negative	0.948 (0.626–1.435)	0.736	43	43
Positive			35	41
Total PTEN expression relative to adjacent non-neoplastic tissue				
Downregulated	0.755 (0.474–1.202)	0.236	43	45
Not downregulated			28	35

Cox regression model was used to investigate the correlation of PTEN expression with overall survival. HR, hazard ratio; CI, confidence interval.

**Table IV:** Correlation of PTEN expression with 3-year survival.

PTEN expression profile	HR (95% CI)	P-value	Median survival time (months)	Survival rate (%)
Cytoplasmic				
Negative	0.697 (0.432–1.124)	0.149	36	57
Positive			28	46
Nuclear				
Negative	1.206 (0.716–2.031)	0.378	36	52
Positive			36	55
Total				
Negative	0.827 (0.522–1.310)	0.520	36	57
Positive			35	49
Total PTEN expression relative to adjacent non-neoplastic tissue				
Downregulated	0.800 (0.480–1.335)	0.421	36	55
Not downregulated			28	48

Cox regression model was used to investigate the correlation of PTEN expression with 3-year survival. HR, hazard ratio; CI, confidence interval.
